# Modeling transformational policy pathways on low growth and negative growth scenarios to assess impacts on socioeconomic development and carbon emissions

**DOI:** 10.1038/s41598-023-42782-y

**Published:** 2023-09-25

**Authors:** Jonathan D. Moyer

**Affiliations:** University of Denver, Denver, USA

**Keywords:** Climate-change impacts, Governance

## Abstract

Degrowth advocates argue for structural transformations in how economies and societies prioritize material wealth accumulation to reduce the negative effects of future anthropogenic climate change. Degrowth proponents argue that human economic activity could be lessened, and societies transformed to prioritize improved wellbeing, reducing the threat of climate change. This paper explores implications of alternative patterns of economic growth with transformational policy pathways (i.e., redistribution) to assess what effects economic growth and broader policies have on changing patterns of human development across both the Global North and South. Using the International Futures model, this article shows that negative growth and societal transformations in the Global North are possible without dramatically damaging long-term global socioeconomic development, though these interventions do not solve the global climate crisis, reducing future cumulative carbon emissions by 10.5% through 2100. On the other hand, a global negative growth scenario will significantly reduce future cumulative carbon emissions (45%) but also dramatically undermines the pursuit of global development goals, like the elimination of poverty. Even with global policies that significantly increase cash transfers to the poor and retired, dramatically improve income inequality, and eliminate military spending, the Global Negative Growth Big Push scenario leads to an increase of 15 percentage points in global extreme poverty by 2100.

## Introduction

*“There is a way of wasting less, eating less, and spending less which gives not less but more.”*^1^.

Present and future challenges posed by anthropogenic climate change are undeniable^2,3^ with well-established effects on natural systems^4–8^. Research shows that climate change will negatively impact socioeconomic development, including by disrupting food security^9,10^, economic activity^11–13^, demography^14,15^, human development^16,17^ and conflict^18–20^. Because of these potentially catastrophic changes to the conditions that support human life, climate change mitigation has been widely studied, with common solutions focusing on investing in renewable energy, improving energy efficiency, and placing a price on carbon^21^. While these policies could lead to a significant reduction in greenhouse gas emissions, they also have been criticized because they almost universally include assumptions about continued economic growth which will force significant technological transformations in energy systems that may be unrealistic^22–25^.

Degrowth^26^^: 195^ research is an important critical perspective that refocuses attention away from a paradigm of continuous economic growth, conspicuous consumption, planned obsolescence, and towards humans living within environmental constraints, particularly the most affluent and largest contributors to climate change^24,25,27–29^. It focuses on an “equitable downscaling of production and consumption that increases human well-being and enhances ecological conditions at the local and global level, in the short and long term^28^.” It emphasizes downscaling of growth as a goal, reducing destructive forms of production and enhancing the public sector with a refocus of economic activity on securing human need^25^.

While previous research has used models to evaluate degrowth for particular countries^23,30^ or to represent biophysical limits to energy production^31^, there has been no research that forecasts changing patterns of economic activities across scenarios over long time horizons on a global and regional basis, exploring how changing patterns of economic growth and shifts in policy strategies impact multidimensional human development as well as carbon emissions^32–34^. How many resources would be available for government spending programs in a world of negative growth?

This article shows that a global negative growth scenario would not provide sufficient material resources to promote global human development, including in scenarios where the global income distribution becomes very equal, military spending is reallocated to other sectors, and government programs significantly increase cash transfers. Fortunately, most degrowth researchers focus on applying these structural transformations in high income countries, recognizing that the rest of the world requires economic activity to promote human development. In a scenario of *High Income Negative Growth* (Table 1) the socioeconomic costs remain high but can be partially offset with dramatic changes to global inequality. In the most ambitious scenario (*High Income Negative Growth Big Push*) poverty is reduced by 377 million by 2050 relative to the *Current Path* and carbon emissions are lessened by 10.9% relative to a *Current Path* development scenario (a similar scenario to SSP3/SSP4^35–37^ and RCP 6.0/4.5^38^). Such a transformation in social organization would be unprecedented, and global shifts in income inequality of that magnitude or an absolute decoupling of long-term economic growth from human development outcomes has never been seen. Additionally, carbon emission reductions in *the High Income Negative Growth* scenario are moderate, as a large share of future emissions will be from countries in other income groupings this century and degrowth reduces resources invested in renewable energy.

The modeling work presented in this article does not build a ground-up degrowth scenario that would be recognizable to many proponents and instead focuses on modeling the broad material needs for socioeconomic development juxtaposed with alternative patterns of carbon emissions across income groups. To that end, this article proceeds by exploring degrowth in more detail, highlighting what measures of economic development are intended to do and what their limitations are, and then proceeding to introduce the methodology and results. The article concludes that the degrowth movement is an important piece of a broad societal response to sustainable human development that is more relevant for already developed regions and that it should be pursued in conjunction with a range of other policies that also promote balanced technological solutions to the problem of human development on a planet of finite resources.

## Background

The degrowth movement emphasizes that continuous economic growth is not possible in a world of finite resources^24,25,27–29,39^, a theme in previous literature modeling long-term societal transitions^40^. The pursuit of economic growth has led to social inequality, as resources and wealth are concentrated in the hands of a few. A degrowth approach requires a planned “scaling down” of economic efforts in areas where general economic wellbeing has been achieved, intending to use both government action and individual choices to intentionally adjust economic activity away from harmful practices that use fossil fuel inputs to one where growth in economic output is not a policy goal while human well-being is^25^.

While there are many policy objectives embedded in the degrowth debate, a central focus is the reduction of the harmful use of fossil fuel and other material resources that are the primary contributor to climate change. Degrowth helps improve climate change by reducing energy demand, effectively undercutting the ability of economic systems to draw upon fossil fuels and use them to project greenhouse gases into the atmosphere. A reduced growth scenario could be important for lowering future carbon emissions and is seen to be one potentially viable pathway to achieving a 1.5 degree centigrade increase in global temperature^24,41^.

Degrowth emphasizes the close connection between energy resource use and economic production^22,42,43^, arguing that “green growth” is largely a mirage and that current energy consumption is unsustainable if the world’s standard of living were raised to that of the Global North. For example, no country has successfully achieved an absolute decoupling of economic systems from energy systems^42^, though there are ample examples of relative decoupling of the rate of growth in economic production from energy consumption, or reducing energy intensity^43^.

Research has emerged that uses quantitative models to evaluate how degrowth might impact human development and environmental outcomes. The MEDEAS model is an integrated assessment model that focuses on the transition to sustainable energy, adding dynamics that show that biophysical limits to energy extraction will lead to a severe global crisis with increased conflict that may collapse modern civilization^31^. This model of degrowth presents an alternative baseline that emphasizes the consequences of stymied future fossil fuel extraction under degrowth.

The EUROGREEN model presents a degrowth alternative for development in France that is compared with a green growth scenario by introducing policies that guarantee jobs, reduce economic growth, grow the public deficit, and lower emissions^23^. The degrowth scenario shows that lowered economic output is possible after multiple decades and can be balanced against core labor inputs, enhancing both environmental outputs as well as human development. LOWGROW SFC is a post-Keynsian demand-driven model for Canada that has been used to analyze policies with outcomes related to sustainable prosperity and environmental burden, finding that a degrowth scenario outperforms a green growth scenario in overall measures of socioeconomic development, driven primarily by the assumed reduction in carbon emissions^30,44^.

Degrowth contains within it a broad critique of measures like GDP often focusing on the limits of using it as a measure of human development or well-being^45,46^. First, GDP growth has no direct relationship with human well-being and can increase while not improving subjective happiness^28^ or other measures of human well-being^47^. Second, GDP can increase with factors that are negatively associated with human well-being, like natural disasters or dramatic shifts in commodity prices. Third, GDP is an incomplete measure when it comes to the treatment of economic inputs and costs, such as climate change and natural resource services. For example, no measure of GDP fully captures the value of key ecosystems for future human development, a key omission when used to study the future of sustainable human development.

Others criticize how particular measures of human development are used, like poverty indicators. First, extreme poverty thresholds such as $1.90 per day (in 2011 dollars) are an absurdly low level of material resources to consider to be viable for a good life. Second, the purchasing power adjustment made to poverty measures has been criticized for introducing measurement error^48–50^. Third, measures of poverty are argued to be imprecise and require frequent household surveys that only capture a particular slice of poverty at a given point in time and do not comprehensively measure poverty.

It is important to disentangle the relationship between GDP and human development by discussing the ways in which GDP as a measure is used. GDP is a measure of the total goods and services produced and consumed by a country and can be measured in various ways. First, it can be measured as the sum of annual value-add produced across sectors in an economy. This is a measure of the total additional value produced by each sector in excess of sectoral investments as measured by a national currency and can be referred to as the “production approach”. Second, GDP can be measured as the total amount of income earned by households and business within a given period (the so-called “income approach”). Alternatively, GDP is also measured by the total expenditures across key actors in the economy, represented in the equation below:

$$GDP=C+G+I+(X-M)$$

where GDP is the sum of C = household consumption, G = Government expenditure, I = Investment, X = Exports, and M = Imports.

Each of these approaches needs to reconcile within a national accounting framework providing a redundant measure that captures various aspects of micro-economic activity in a single model.

GDP per capita is the per-person share of the total economic activity of a country and is occasionally used as a proxy measure for individual incomes, but this is misleading. GDP per capita and household or individual incomes are not connected directly, though the two indicators do have a positive linear relationship. GDP per capita is a better measure of the relative “sophistication” of an economy especially when controlling for the value of commodity and extractive resources, which tend to suffer from rent-seeking type characteristics. GDP per capita can be used as an input into models that measure things like household consumption but should not be used directly to capture household consumption as they have no relationship with the distribution of resources in a society. Finally, GDP growth is the year-to-year (or period-to-period) change in total GDP. Growth in the overall size of economic activity is the namesake of the “degrowth” movement and is the factor that is meant to be provocatively contained, controlled and managed.

GDP can be measured at market exchange rates (MER) or purchasing power parity (PPP), both of which provide different insights into how broader economic activity is valued. MER measures capture a nation’s economic activity and value it based on a national currency relative to other national currencies, or one benchmark currency. Alternatively, PPP measures inflate or deflate GDP at MER values based on a basket of comparable goods. GDP is also often measured in “real” currency which controls for inflation across time. Real currency conversions should be compared with “current” currency, in which inflation is not controlled for. Most measures of poverty use real PPP adjusted income in fixed US dollars.

Degrowth researchers often focus on the negative outcomes associated with economic growth and the shortcomings of general measures of economic production/consumption, like GDP. But economic growth changes the underlying material resources available for development and is an important aspect of development, be it controlled by the private sector or the state. Economic growth increases production which makes more resources available for consumption, a key determinant of household income and poverty^51–54^. Economic growth also directly feeds government revenue and allows for spending on public services, investment in future technology like renewable energy, as well as providing resources for key determinants of human development like healthcare spending, education spending, infrastructure, etc. And, while the measurement of economic growth and activity is imperfect, it remains a useful tool for understanding economic activity and joins a range of other measures capturing socioeconomic progress that all have clear limitations, though are used in research supporting degrowth and other policy solutions.

## Methodology

I use an established integrated assessment modeling framework that represents human, social and natural system development called International Futures (IFs) to analyze the implications of changing patterns of economic growth on human development (See model documentation here: https://pardeewiki.du.edu/index.php?title=Understand_the_Model). I present a series of scenarios listed in Table 1. These scenarios are then evaluated across various dimensions of human well-being to evaluate broad trade-offs in achieving sustainable human development. These scenarios were created for the globe as well as all World Bank income groupings, though the main text of the article focuses on the globe and High Income groupings. The Appendix reports results for other income groups and the replication instructions can be used to reproduce results for individual countries (Replication found here: https://ifs02.du.edu/Replication%20Files/DeGrowth%20sce.zip).

**Table 1:** Description of the scenario components used in this analysis.

Scenario name	Intervention
Current path	A dynamic scenario that forecasts within and across systems for 186 countries through 2100. The scenario results behave most similarly to SSP3 or SSP4 for variables measuring economic change and demographics
No growth	GDP growth reduced to zero from 2018 to 2100
Negative growth	GDP growth reduced to negative one from 2018 to 2100
Military spending	Reduced to near zero from 2018 to 2100 for the world, spending on education, health and infrastructure protected
Government transfers	Increased five-fold for both pension and welfare
Inequality	Reduced to 0.2, the lowest current measured level of income inequality on the Gini scale (Albania)
Big push	Combining the interventions for *Military Spending*, *Government Transfers*, and *Inequality*

The IFs model^55^ is an integrated assessment tool that includes the following dynamically interconnected systems: agriculture^56^, climate^57,58^, conflict^59^, demographics^55^, economics^60,61^, education^62^, energy^63^, gender^64^, governance^65^, health^66–68^, infrastructure^69^, international relations^70,71^, poverty^53,72^, and technology^73^. IFs can be used to explore broad questions of integrated development within and across systems and forecasts variables for 186 countries from 2017 to 2100. The data used as inputs into the model come from a wide range of sources^74–81^.

The analysis presented here relies on multiple scenarios that vary core assumptions related to a) the future of economic growth by country group; b) the future of cash transfers from governments for both welfare and pension; c) changing patterns of income distribution (as measured by the Gini coefficient for income inequality); and d) changing patterns of government spending, particularly on the military. Each of these alternative scenarios is compared with a *Current Path* scenario that attempts to capture a dynamic future path that is “most likely” considering the data and factors used within the IFs system.

The core models in the *Current Path* scenario include demographics, an agent-cohort-component model with endogenous representations of births, deaths and migration^55^. The economic module is built on a Cobb–Douglas production function with Solow residual broken down across different areas of productivity contribution and including a social accounting matrix (with an embedded input–output table measuring activity across six sectors)^60^. It includes an education model representing schooling by sex across primary, lower secondary, upper secondary, tertiary and vocational education with variables representing math and reading quality^62^. The health system has both distal and proximate drivers represented that forecast mortality and morbidity by age and sex for 15 different categories^66,67^. The infrastructure module represents ICT access, electricity production and access, water and sanitation access as well as road construction and access^82^. The governance module includes forecasts of security, capacity and inclusion^65^. Agriculture is modeled using a partial equilibrium tool that includes a land-use module and a technology module^56^. Energy is also represented using a partial equilibrium tool that includes the production and consumption of energy by type across sectors like oil, gas, coal, renewable, and nuclear^83^. The environmental module includes a carbon cycle as well as impacts from changing CO2 parts per million in the atmosphere, temperature and precipitation^73,84^. Both gender and technology are cross-cutting components of the model^64^.

The effect of economic growth on human development is a key component of this analysis. Economic growth changes patterns of human development in various ways in the IFs model. First, and most directly, GDP growth increases the total amount of economic resources available to various actors throughout the model (actors, governments, firms). Second, and more importantly, GDP per capita has broad impacts across various aspects of the model. Average production per capita changes demand for goods and services across sectors, impacts household consumption preferences, and changes the productivity model boosting demand for government services like health and education. GDP per capita also has a relationship with household consumption mediated by various factors^53^. Changing GDP per capita is a distal driver of long-term projections of intrastate conflict, societal values like inclusive governance, and other particular outcomes that reflect broad social transformations.

GDP growth is not a panacea for development in IFs (or the real world). GDP growth increases carbon emissions when economies are based on fossil fuel energy consumption. The IFs model represents this endogenously across fossil fuel sources including coal, natural gas and oil. As carbon emissions increase they change the accumulation of greenhouse gas in the atmosphere which changes patterns of precipitation and temperature along with having a broad and negative impact on economic production through “damage functions”^85,86^.

The scenario interventions also simulate transformations in how income is distributed, the volume of government cash transfers for welfare and pension, as well as a transformation in how governments allocate resources away from the military and towards systems that are more direct investments in long-term human development. The treatment of inequality in the IFs model starts with the Gini coefficient for income inequality, derived from the Lorenz Curve, and described in literature^53^.

Government transfers and military spending patterns are both represented within the government finance module of IFs. First, IFs conceptualizes government finance by modeling government revenue as a function of taxation and foreign aid. Next, IFs calculates the demand for government spending across areas of consumption (e.g., education, health, infrastructure, security) and transfers (e.g., welfare and pensions). IFs balances these government spending sectors dynamically across time and reconciles revenue and expenditures/transfers with all other global financial patterns through a social accounting matrix^55^.

The final submodule of the IFs model used in this analysis is the energy module as a driver of carbon emissions. The energy module is a partial equilibrium model that represents ultimate recoverable resources, available reserves, and production for coal, natural gas, oil, hydro, nuclear and a general category of renewable energy that includes wind and solar. The model “chases equilibrium” between supply and demand for energy, relies on a global energy price to mediate these dynamics, and includes energy trade. The energy module feeds the carbon emissions model where weights to note the carbon intensity of different fuel types are used to calculate overall emissions. The model includes the ability to adjust investment-to-output, efficiency, and production levels across energy type by country or region as well as a broader carbon model that calculates overall carbon concentration in the atmosphere.

## Results: *Current Path*

**Figure 1:**
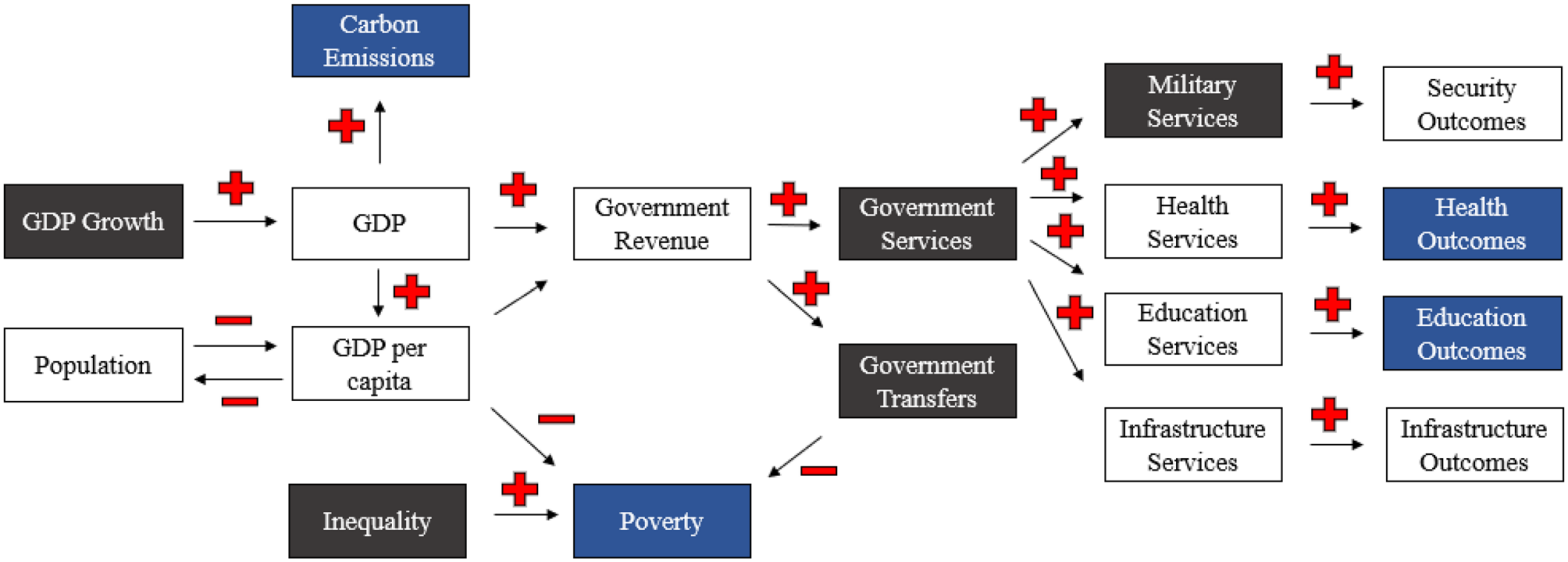
Conceptual Overview of Variables in IFs used in this analysis. Black boxes represent variables used in scenarios and blue variables represent key outcomes reported. The plus and minus signs reflect the direction of the relationship. These variables are a small sub-set of the broader model.

**Figure 2:**
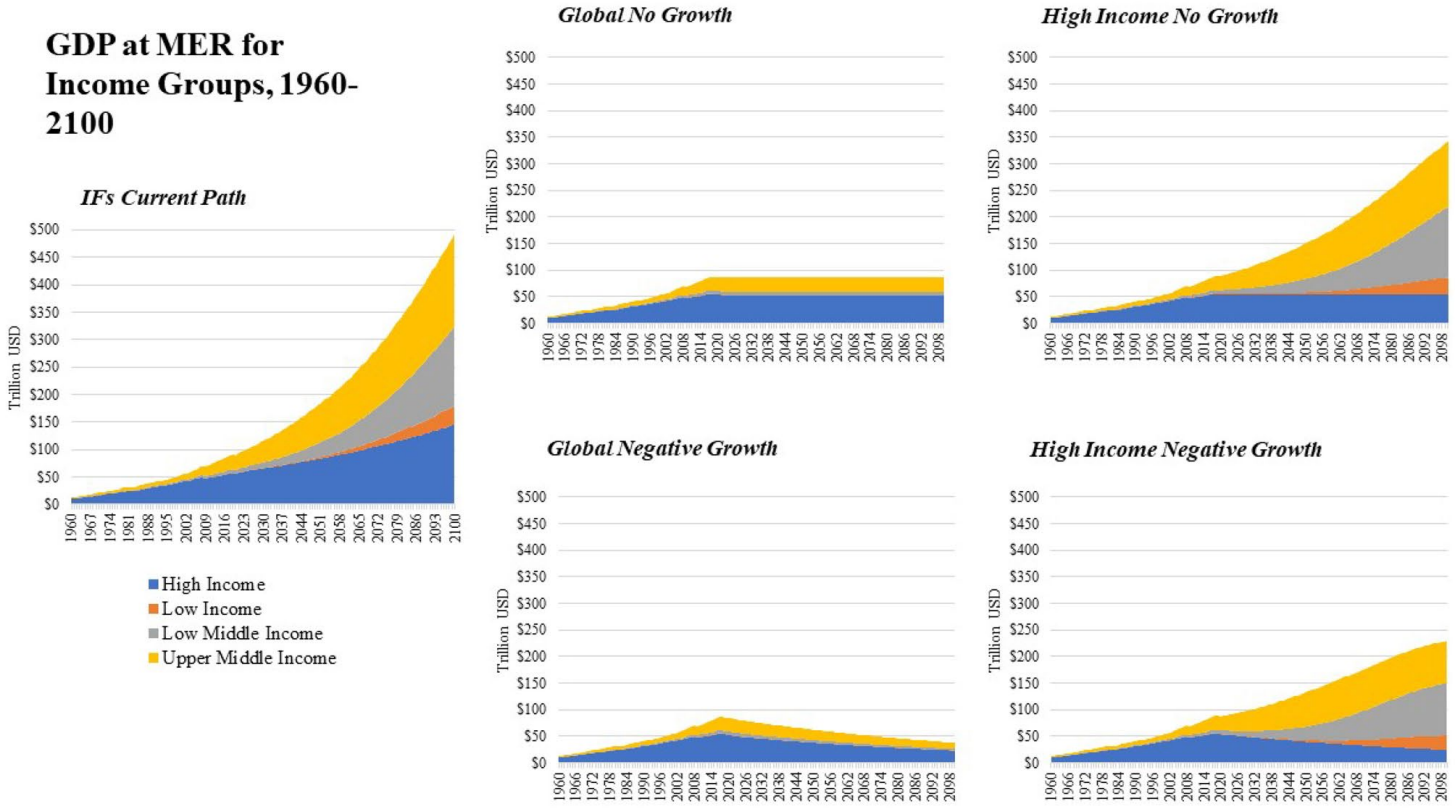
GDP at MER from 1960 to 2100 by Income Group and Scenario.

The IFs *Current Path* scenario forecasts global economic growth measured by GDP at MER to average 2.3% by 2030 and then decline to under two percent through the end of the twenty-first century. Compared with other long-term economic growth forecasts, this projection is most similar to the economic projections in SSP3 or SSP4^35,61^, two scenarios that are pessimistic about future socioeconomic development (see Appendix and Figs. 1 and 2). Over fifty percent of all economic activity through mid-century will occur in High Income economies and over thirty percent of all economic activity will be from High Income economies by the end of the century along the *Current Path*.

**Figure 3:**
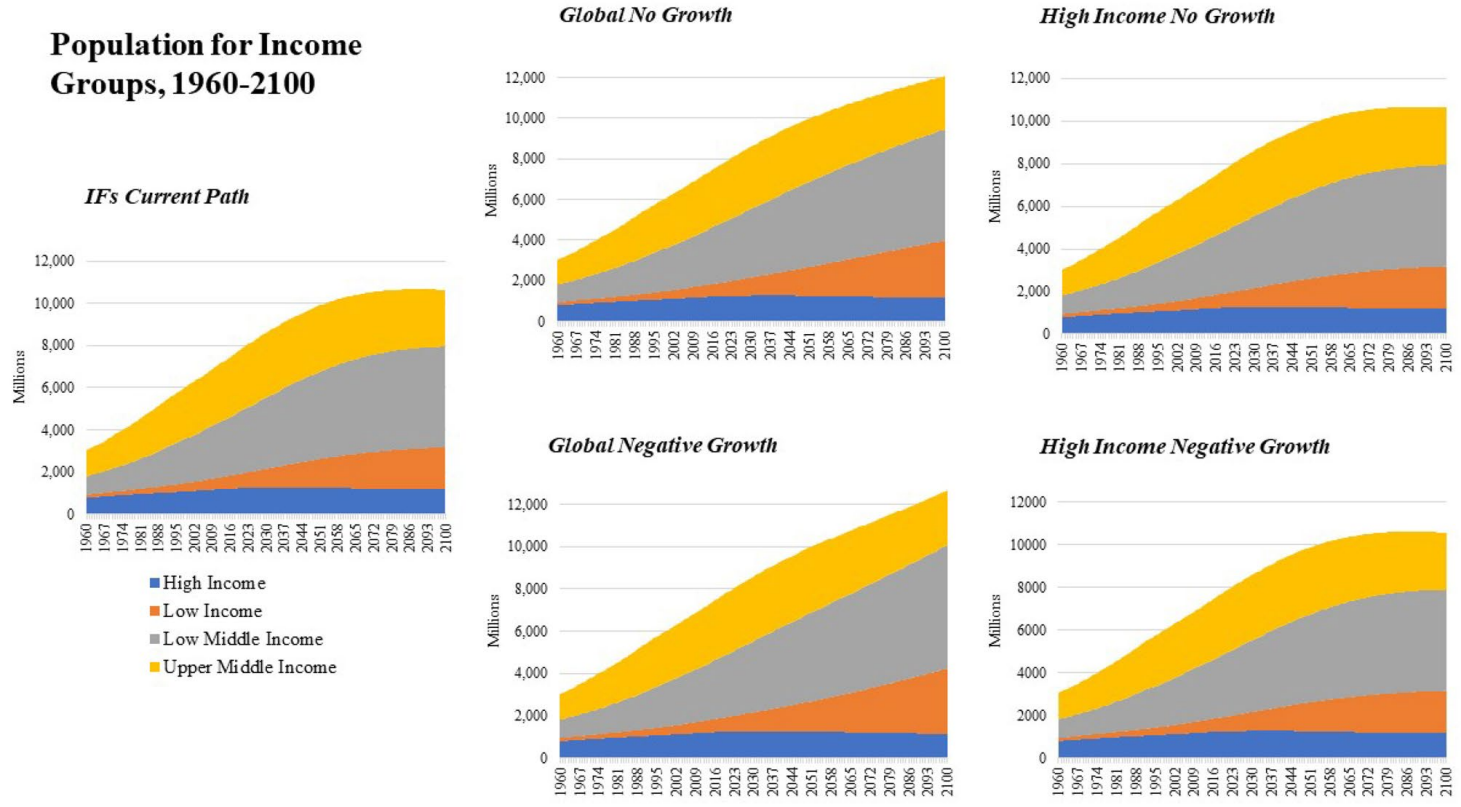
Population from 1960 to 2100 by Income Group and Scenario.

**Figure 4:**
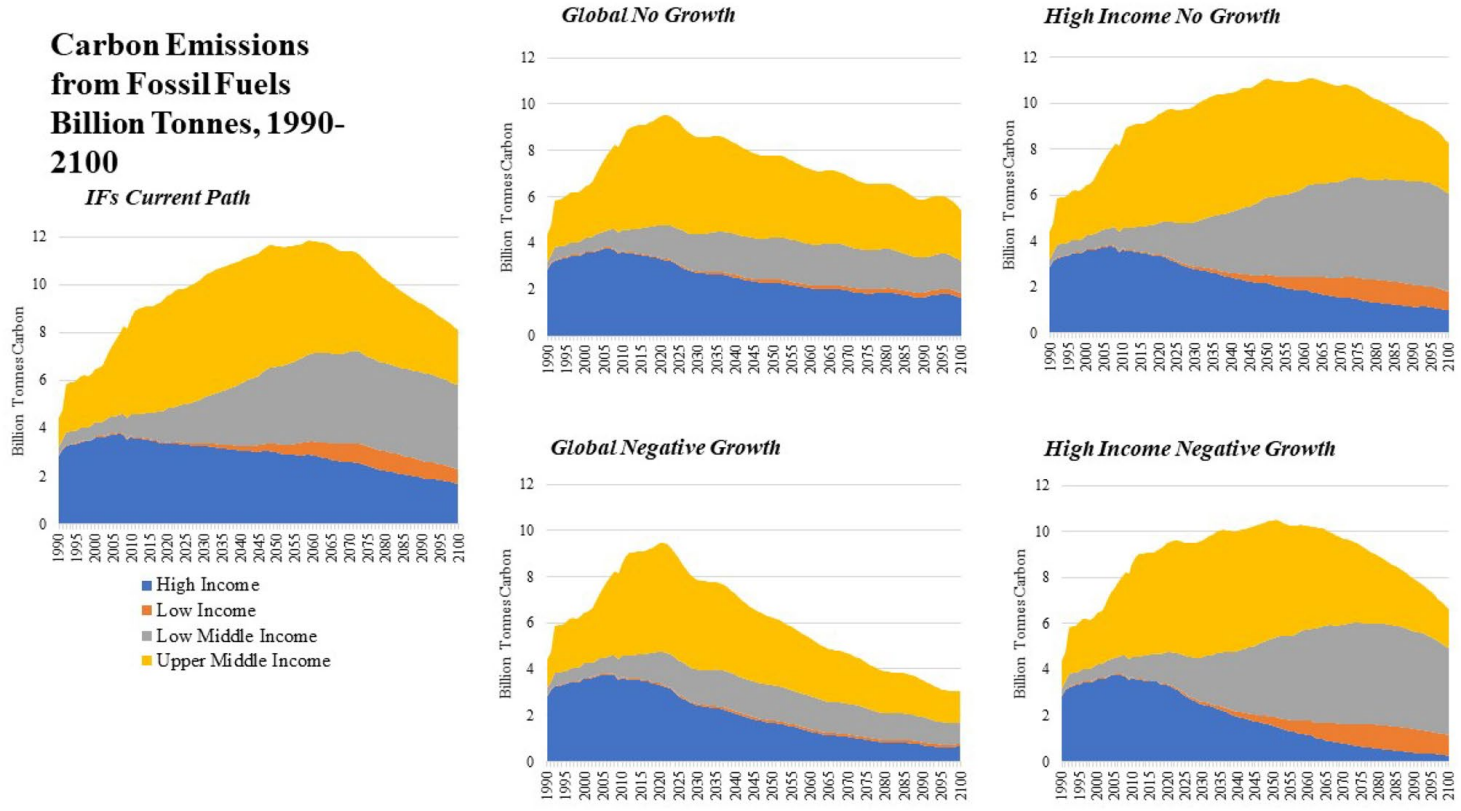
Carbon Emissions from Fossil Fuel use in Billion Tonnes, 1990–2100 by Income Group and Scenario.

The *Current Path* projects that global population (Fig. 3) will surpass ten billion near mid-century with the majority of growth occurring in Low-Middle Income economies (Fig. 4). This is similar to the UN Population Division medium variant and SSP3^36^. While High Income economies represent more than 50% of future economic activity through mid-century, they are projected to only account for 12.8% of global population by mid-century.

Historically, while representing a small share of the global population, High Income economies were responsible for 48.6% of total carbon emissions from fossil fuel usage from 1990–2017. Upper-Middle Income countries also contributed significantly to carbon-based fossil fuel usage over that period, emitting 40.8% of carbon from 1990 to 2017, a proportion that was more closely aligned with their population share (see Figs. 3 and 4). Over that same period Low-Middle Income countries emitted 9.8% of global carbon from fossil fuel usage. In the *Current Path* scenario, the majority of future emissions from fossil fuels come from Upper-Middle Income economies, which are projected to emit 47.5% of global carbon emissions through mid-century followed by High-Income economies which are projected to emit 30.2% of carbon emissions. Low-Middle Income economies are projected to emit 20.7% of carbon emissions through 2050. Low-Income economies are projected to emit slightly more than 1.5% of carbon emissions through mid-century.

**Figure 5:**
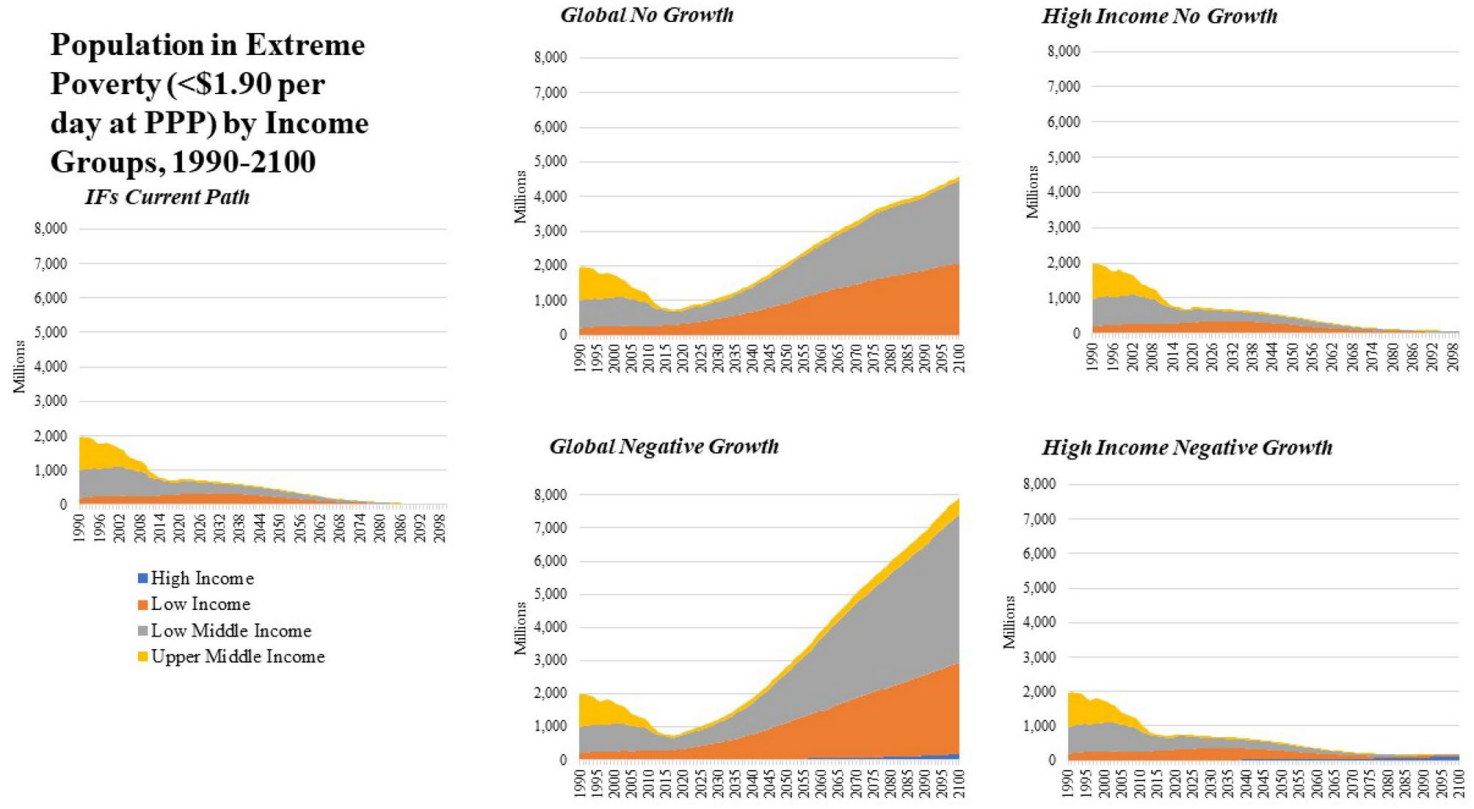
Extreme Poverty (< $1.90 per day at PPP) by Income Group and Scenario.

Turning to extreme poverty, the majority of the world’s poor currently live in Low-Middle Income and Low-Income countries which represent over ninety percent of the world’s population living on less than $1.90 per day (Fig. 5). The *Current Path* projects that global extreme poverty will remain a persistent problem with more than 500 million people living under that threshold through mid-century.

**Figure 6:**
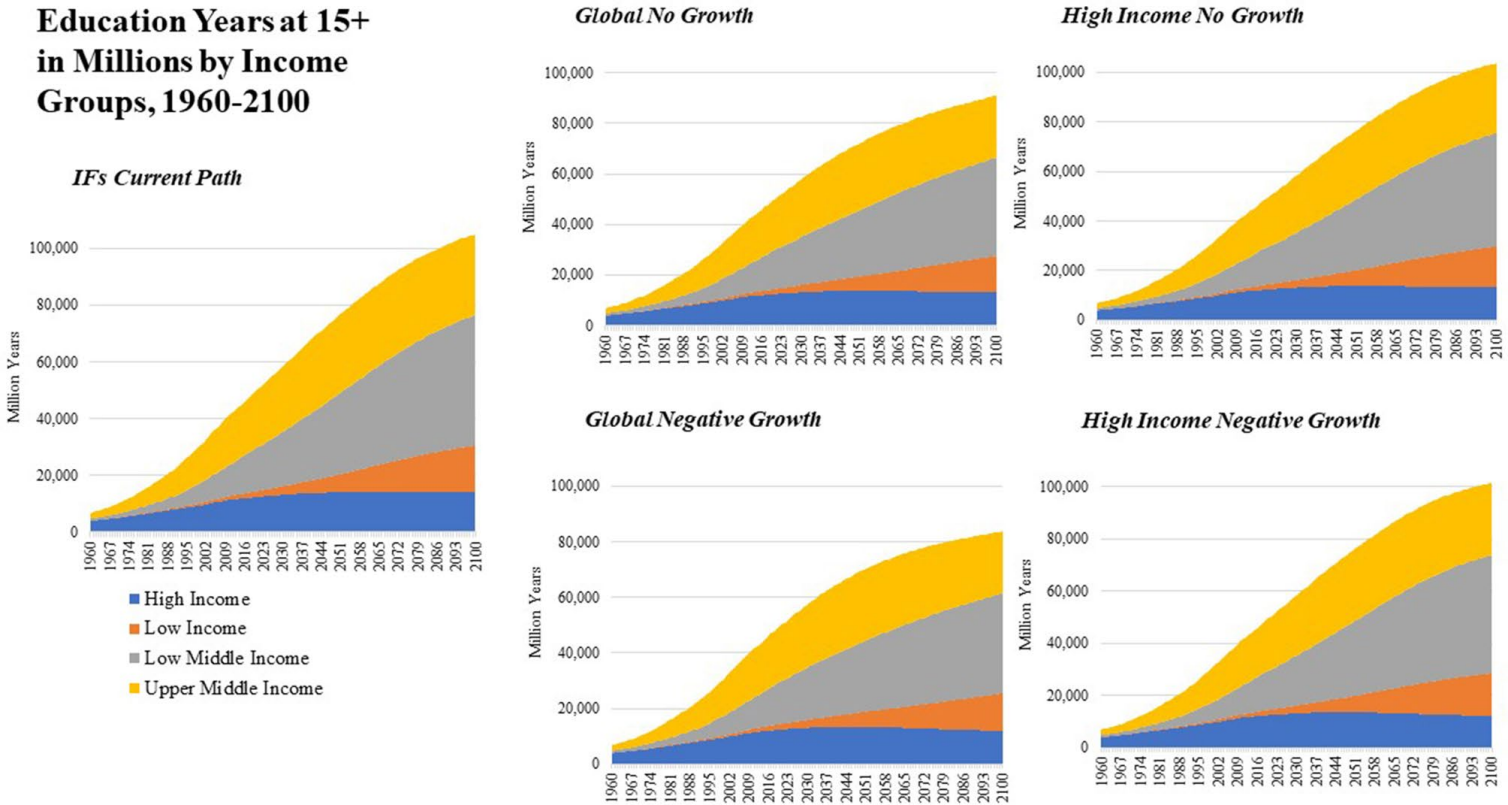
Years of Education at 15 + , 1960–2100 by Income Group and Scenario.

**Figure 7:**
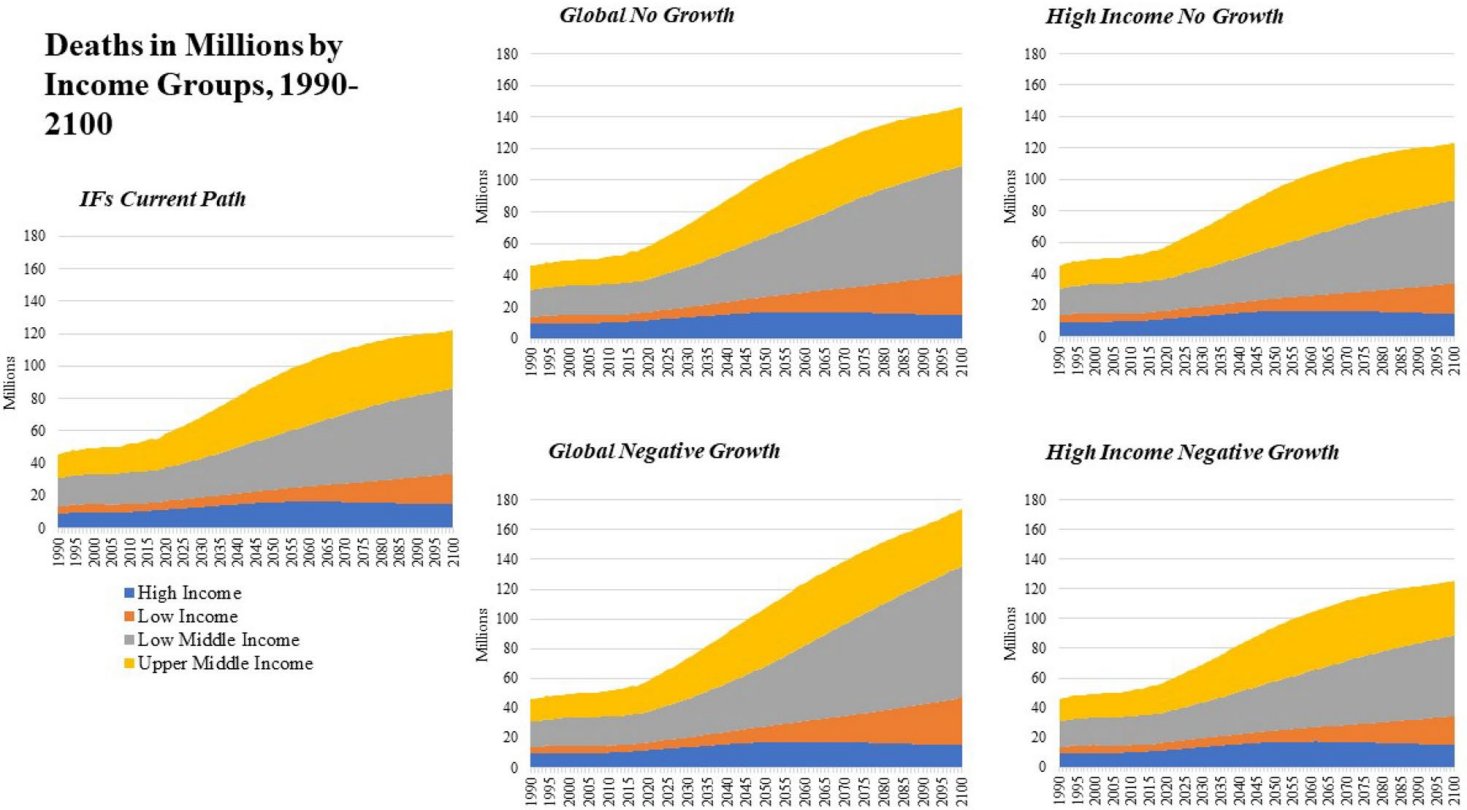
Deaths in Millions from 1990 to 2100 by Income Group and Scenario.

Broader measures of human development that move beyond consumption-based poverty that are modeled in IFs include average life expectancy and years of educational attainment, among others (Figs. 6 and 7). The IFs *Current Path* scenario projects that life expectancy in High Income countries will continue to grow from over 80 years today to 83.7 years by mid-century. Across other income groups life expectancy gains are projected to converge to High Income levels, which growth in the frontier of life expectancy is expected to see diminishing marginal improvements to additional investment. The same is true for advances in education and summary measures included in IFs like the Human Development Index.

IFs models government spending it in two ways. First, the model projects spending on services that includes the following sectors: Military, Health, Education, R&D, Infrastructure, and Administrative. In 2017, total global government spending on these services exceeded $16 trillion, with the majority of spending on health ($5.1 trillion) followed by education ($4.1 trillion), infrastructure ($3.0 trillion) military ($1.9 trillion), administration ($1.8 trillion), and R&D ($215 billion). The *Current Path* projects that total government consumption on these services will grow to $19.7 trillion by 2030 and $31.9 trillion by 2050.

IFs also models government transfers to both pension and welfare. In 2017, IFs estimates that government transfers for both welfare and pensions exceeded $14 trillion, with High Income economies representing nearly $10 trillion of those total transfers. Through 2030, IFs forecasts these to increase to $18.9 trillion by 2030 and $31.9 trillion by 2050. By 2050, High Income economies are forecast to represent $16 trillion of that total, while Upper-Middle Income economies represent $12.6 trillion of government cash transfers in the *Current Path* scenario.

### High income no growth scenarios

The *High Income No Growth* scenario keeps High Income economic activity at $55 trillion per year from 2017 to 2100. The cumulative reduction in economic activity exceeds $68 trillion by 2030, $449 trillion by 2050, and $3,298 trillion by 2100 relative to the *Current Path*. High Income population stands at 1.22 billion people in 2017 and was projected to peak at 1.27 billion by 2040 in a *Current Path* world. In the *High Income No Growth* world, population growth peaks slightly earlier and at slightly lower levels driven by higher overall mortality associated with less government spending on health. GDP per capita (measured in PPP terms) remains flat between $42 and $47 thousand per person throughout the century, varying as population levels change.

The *High Income No Growth* scenario reduces the amount High Income governments can spend on services from $11.3 trillion in 2017 to $10.04 trillion by 2030 and $10.4 trillion by 2050. This represents a cumulative reduction in spending across that period of $16.9 trillion by 2030 and $101.5 trillion by 2050. The majority of those reductions occur in health (by $5.5 trillion by 2030 and $37.3 trillion by 2050), education (by $4.5 trillion by 2030 and $37.3 trillion by 2050), infrastructure (by $2.5 trillion by 2030 and $13.5 trillion by 2050), military (by $2.2 trillion by 2030 and $26.2 trillion by 2050). Government to household transfers for welfare and pensions in High Income countries are estimated to be $10.0 trillion in 2017, a figure projected to increase to $11.8 trillion by 2030 and $16.1 trillion by 2050 in the *Current Path*. In the *High Income No Growth* scenario the level of government transfer reduces to $9.8 trillion by 2030 and $10.7 trillion by 2050. This represents a cumulative reduction in transfers of $12.7 trillion by 2030 and $86.0 trillion by 2050.

The effects of these reductions in economic activity and government spending/transfers is to slow, but not eliminate, progress across human development indicators measuring cumulative health and educational outcomes in High Income countries. Life expectancy was projected to grow in the *Current Path* from 80.4 years in 2017 to 83.7 years by 2050. Lower spending reduces average life expectancy to 82.7 years by mid-century, driven by more rapid increases in annual deaths, which were forecast to grow in a *Current Path* scenario from 10.9 million per year in 2017 to 16.0 million per year by mid-century, driven primarily by rapidly aging populations. In a *High Income No Growth* world, these deaths increase to 16.4 million by mid-century, a cumulative increase of 10.8 million deaths over that period of time. The majority increased deaths are cardiovascular, projected to be 17.9% higher in a *High Income No Growth* scenario compared with the *Current Path*^66^.

Average years of education for the population age 15 and older were projected to increase in a *Current Path* scenario from 11.8 years in 2017 to 13.0 years by 2050 in High Income countries. In a *High Income No Growth* scenario, this increase is slightly slower, at 12.8 years by mid-century. The percent of adults who complete tertiary education is projected to grow in a *Current Path* world as well as a *High Income No Growth* world, though with less growth in spending per student and less growth in levels of quality, measured in terms of reading and writing scores across level and sex.

Levels of poverty increase in a *High Income No Growth* scenario relative to the *Current Path*. IFs estimates that the number of people living on less than $1.90 in High Income economies was 7.2 million in 2017 and was projected to decline to 1.5 million by 2050. In a scenario of no growth, this is projected to increase to 9 million by 2030 and 13 million by 2050. This represents an increase of 4.6 million people in extreme poverty in High Income countries by 2030 and 11.4 million by 2050.

In the *Current Path*, imports and exports are projected to increase from $31.3 trillion in 2017 to $57.5 trillion by 2050 between High Income countries and the rest of the world. In the *High Income No Growth* scenario, growth in imports and exports stagnates and remains at levels between $34 and $37 billion across the time horizon. Cumulatively this accounts for $325.8 billion by mid-century relative to the *Current Path*. In 2017, IFs estimates that High Income countries provided $125.6 billion in foreign aid, a number that is projected to increase in a *Current Path* world to $182.9 billion by 2050. In a *High Income No Growth* scenario the level of foreign assistance remains at $125.8 billion through 2050 representing a cumulative reduction in aid of $912.9 billion through mid-century. Stocks of foreign investment in High Income countries is also reduced. IFs estimates that foreign investment in 2017 was $28.5 trillion and projected to grow to $60.7 trillion by 2050. The *High Income No Growth* scenario shows growth in foreign investment as well, just at lower levels. Here investment increases to $34.9 trillion by 2030 and $43.1 trillion by 2050. IFs estimates remittances flowing out of High-Income countries in 2017 to be $350.9 billion a number that grows to $487.3 billion by 2030 and $852 billion by 2050 in the *Current Path*. In the *High Income No Growth* scenario, that growth is slower, increasing to $443.2 billion by 2030 and $687 billion by 2050, representing a cumulative reduction of $2.28 trillion in global remittances by mid-century.

The impacts of these changes on development in other countries can also be seen. For example, in a *High Income No Growth* scenario, global extreme poverty increases by 10.1 million people by 2030 (inclusive of the 4.6 million increase in High Income countries reported above) and 34.5 million by 2050. Other effects come more slowly through changing patterns of economic interdependence. For example, Chinese GDP is reduced cumulatively by $15.4 trillion by 2050 because of reduced exports to High Income countries. Other countries that are particularly dependent on foreign aid experience down-turns. Uganda, for example, is projected to increase its receipts of foreign aid from $2.4 billion in 2017 to $7 billion by 2050 in the *Current Path*. In a *High Income No Growth* scenario they see a cumulative reduction in aid of $3.1 billion by 2030 and $28.6 by 2050. This leads to an increase of 3.5 million people in extreme poverty in Uganda by 2050, effects that can be seen in a variety of Low Income countries.

The *High Income No Growth* scenario does reduce carbon emissions from fossil fuel sources. In 2017 High Income economies emitted 3.4 billion tonnes of carbon, a number projected to decline to 3.0 billion tonnes by 2050 in the *Current Path* scenario. By eliminating growth, carbon emissions reduce to 2.8 billion tonnes by 2030 and 2.2 billion by 2050. This represents a cumulative reduction of 16.05 billion tonnes from 2017 to 2050, or a 15.3% reduction in emissions in High Income economies (a 3.6% global reduction).

Part of the reason that the effect on carbon emissions is more limited than might be expected is because investments in renewable energy are constrained because of fewer overall material resources that can be invested in improving and distributing technology. Overall energy investment in High Income economies stood at $332.3 billion in 2017 and was projected to grow to $378.8 billion by 2050. The cumulative reduction in investment across this period of time is $2.0 trillion, or -15.8% relative to the *Current Path*. This leads to slower growth in renewable energy, which increases from 1.1 billion barrel of oil equivalent (BBOE) in 2017 to 3.7 BBOE in the *High Income No Growth* scenario compared to 4.7 BBOE by 2050 in the *Current Path*, a reduction of 11.7%.

By adding *Big Push* policies to the *High Income No Growth* scenario shows that some strategies are particularly effective in mitigating socioeconomic costs while others are more prone to developmental trade-offs^87,88^. A global scenario where income inequality is reduced to the levels of the best performing countries in 2017 does eliminate poverty in the *High Income No Growth* scenario through 2100. Other *Big Push* interventions do not fare as well. Eliminating military spending can off-set reductions in education and health spending, but the *Current Path* shows more education and health spending without eliminating military spending before mid-century. Increasing government transfers can reduce poverty in the short-run, but this comes at the cost of investments in other areas like education and health and growth in the *Current Path* overtakes household transfers in the *High Income No Growth Big Push* scenario before mid-century.

A *No Growth* scenario in High Income countries may be possible, though would require a broad transformation of society (the *Big Push*), a significant centralization of state power and transformations of how every economic sector functions. This would come with significant costs in the short and long-run, some of which can be anticipated and others that are not known. In addition, the long-term benefits in terms of carbon emissions reduction are not so significant that the long-term trajectory of climate change is altered to achieve sustainable levels.

### High income negative growth scenarios

A *High Income Negative Growth* scenario produces results with more amplified findings compared with the *High Income No Growth* scenario. Table 2 presents these findings along with other scenarios described in this article. By 2050, a *High Income Negative Growth* scenario leads to a cumulative lowering of GDP relative to the *Current Path* of $760 billion, a 26.4% reduction. This lost economic activity grows to over $7,000 trillion by 2100, a reduction of 52.6% compared with the *Current Path*. This scenario increases global poverty slightly (by 0.8 percentage points in 2050 and 1.6 in 2100) relative to the *Current Path*), slightly reduces global life expectancy (by 0.4% in 2050 and 1.6% in 2100) and lowers global educational attainment (0.9% in 2050 and 3.6% in 2100). This scenario reduces cumulative global carbon emissions between 2017–2050 by 6.3% and 10.5% through 2100.

**Table 2:**
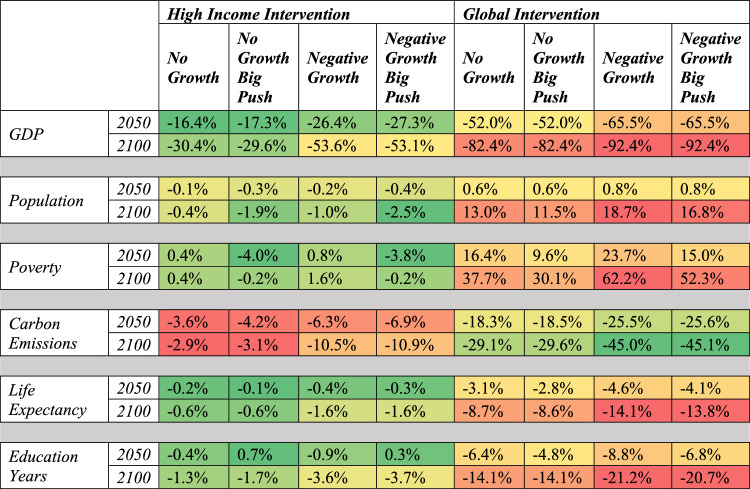
Summary of results for main scenarios compared with the IFs Current Path for 2050 and 2100 across key indicators for global measures of development. Values are color-coded by indicator to highlight general patterns of development and show differences across scenario.

Percentages below represent the following by indicator: a) GDP is cumulative percent difference from 2017-X relative to the Current Path; b) Population is the percent difference in year X relative to the Current Path; c) Poverty is the percentage point difference in year X relative to the Current Path; d) Carbon Emissions are the cumulative percent difference between 2017-X relative to the Current Path; e) Life Expectancy is the percent difference in year X relative to the Current Path; and f) Education Years is the percent difference in year X relative to the Current Path.

A *High Income Negative Growth Big Push* scenario improves global poverty through 2050 by 3.8 percentage points (377.2 million people) and through 2100 by 0.2 percentage points (21.5 million people) compared with the *Current Path*, positive outcomes that stem from the significant assumptions about improved income inequality. There are also slight improvements to overall life expectancy and educational outcomes relative to the *High Income Negative Growth* world, though not relative to the *Current Path*.

### Global no growth and negative growth scenarios

A *Global No Growth* scenario would lead to 1.05 billion people living on less than $1.90 per day by 2030 and 2.08 billion by 2050 relative to the *Current Path*. By 2050 and compared with the *Current Path*, life expectancy in a *Global No Growth* scenario is projected to be 1.2 years lower in High Income countries, 1.7 years lower in Upper-Middle Income Countries, 3.0 years lower in Low-Middle Income Countries and 3.0 years lower in Low Income Countries. Global population is projected to peak at the end of the century at over 12 billion people because of reduced development outcomes pushing fertility rates higher than in a *Current Path* world.

A *Global No Growth Big Push* scenario shows that global poverty would be less than the 2017 value through the year 2035 because of massive investments in government transfers and improved income equality. However, the long-term effects of a lack of resource growth with a growing global population leads to an increase in global extreme poverty which surpasses 1.5 billion by 2050. Re-allocating global military spending to education, health and infrastructure sectors can also delay some of the cost of the *Global No Growth* scenario. For example, in the *Current Path*, global health spending in 2017 is $5.1 trillion and is forecast to grow to $6.6 trillion by 2030 and $11.3 trillion by 2050. A *Global No Growth* scenario sees much less growth in health spending, which is $5.9 trillion in 2030 and $5.8 trillion by 2050. In a world where military spending is reallocated across multiple spending categories, IFs calculates that health spending could increase to $5.8 trillion by 2018 and grow to $6.2 trillion by 2030. However, because of a lack of global expansion of material resources, global health spending grows to $8.2 trillion by 2050, far less than $11.3 trillion in a *Current Path* world.

The benefit of a *Global No Growth* world is seen clearly in annual carbon emissions from fossil fuels. Carbon emissions peak in the 2020s and decline more rapidly across time. This leads to a lower stock of greenhouse gas in the atmosphere (reducing from 527.9 in the *Current Path* in 2050 to 497.3 in the *Global No Growth* scenario and from 700.3 to 589.1 in 2100). This would lead to a lower global average temperature change of nearly one degree centigrade by the end of the century, though also far from sustainable levels.

The most dramatic impact on socioeconomic outcomes is seen in the *Global Negative Growth* scenario which shows an increase in the total number of people living in poverty relative to the *Current Path* of 2.4 billion by 2050 and 7.9 billion by 2100. Life expectancy is reduced by 4.6% in 2050 and 11.7% in 2100 and education years is reduced by 8.8% in 2050 and 21.2% by 2100. Carbon emissions are also dramatically reduced, by 25.5% in 2050 and 45% by 2100 as well. When the *Global Negative Growth* scenario is coupled with the *Big Push* interventions, outcomes improve slightly, but the loss of available resources translates into dramatic reductions in human development even with dramatic transformations in government spending and global inequality.

## Discussion

Degrowth research highlights several important challenges to current global paradigms of sustainable development, issues with measurement, and more fundamental questions about how to live a good life in a way that also promotes sustainable human activity that does not exceed global planetary boundaries. As human welfare and population sizes expand, their demand for energy resources will continue to grow—the degrowth proponents are correct that there can never be an absolute decoupling of energy consumption from economic activity^42^.

But economic activity is important for human development, especially with the expanding global population and real socioeconomic development needs across the Global South. Material resources are the backbone of infrastructure, education, health, governance, security, and the other inputs into multidimensional human development. Degrowth advocates are correct to note that there are decreasing marginal returns to human well-being for additional economic output, especially in high income countries. But this does not obviate the need for material resources for human development. And, while degrowth researchers are correct to note that the Global North has historically benefited significantly from practices of colonialist exploitation and more covert patterns of material resource extraction at the expense of the Global South, current economic interdependence and can be used to benefit the Global South if pursued appropriately^89^. And economic decoupling from fossil fuels systems can also bring about additional costs in terms of increased probability of international conflict^90,91^, impeded global norm development, and nationalism in place of cosmopolitanism as system end-states.

Degrowth also raises important critiques of the character of economic systems and how humans engage with them in the pursuit of happiness, pointing to weaknesses in how our current global economic system promotes well-being. See the very useful critique of consumer capitalism in Büchs and Koch, for example^47^. Profit seeking modes of production can indeed be exploitative of labor domestically and abroad, and history is rife with examples of how global capitalism has created significant impediments to people living their best lives, has created structural imbalances and inequalities that must be addressed, and has increased inequality. It is also clear that capitalism has been an economic system that has created wealth and driven the development of new technologies that have also benefited human development. It is outside the scope of this article to provide an authoritative evaluation of the long-term effectiveness of alternative economic systems, but I do note that most forms of economic production and consumption today are mediated by state authorities and actors that have the ability to regulate unmoored market activity^92^. Global economic activity is a balance between state and private enterprise that produce material resources with differing degrees of control, one over the other across time^93,94^. Whether these resources are distributed equitably or essential services are provided is largely a product of the kind state control in question.

Degrowth is a radical call for change in light of the various crises created by economic expansions on a resource constrained planet^26^. It is meant to provide us with a new way of thinking about what the future could look like beyond the *Current Path*. This analysis implicitly highlights large structural imbalances in the distribution of global resources (the *High Income No Growth* scenario shows that rich countries could simply stop growing and still see improvements in core development outcomes). Imagining a system beyond is indeed a valuable activity and reducing consumption/production patterns may be one say to help solve global environmental issues, though this research shows it is far from a panacea.

Some might profoundly disagree with the analysis presented here because it does not explicitly recognize that global development is in “overshoot” if we do not stop climate change and other ecological crises immediately^95^. These critics point to the dire projections in RCP 8.5, the findings of recent IPCC and IPBES reports that highlight how human development is fundamentally changing the planet in permanent ways, and that humans are toying with natural systems in ways that are, at the very least, incredibly risky and, at worse, immoral and negligent. A *Global No Growth* strategy may be the most optimal choice if the world is on a collision course with planetary boundaries that is unavoidable. However, because ecological systems are characterized by complexity, planetary boundaries are not objective thresholds. Because the socioeconomic costs of a *Global No Growth* strategy are indeed significant and recent research suggests that the most extreme climate scenarios are unlikely^96^, though tipping points and climate extreme scenarios remain under studied^97,98^.

The analysis presented here shows that a *High Income No Growth* scenario will not achieve a profound change in the trajectory of global climate change. Additional interventions will be needed, particularly those that focus on improving our ability to produce and consume using renewable technologies. While these investments will indeed create new challenges, they represent the only viable way forward to achieve reduced climate uncertainty while also promoting human well-being. Some authors have dismissed the role of technology in improving our ability to address significant issues of climate change and human development, arguing that unless we achieve absolute decoupling of energy production and economic growth, human development will always be in tension with the environment^42^. Human development will always require energy and impact the environment, and, barring rapid depopulation, the Anthropocene is here for the foreseeable future.

Degrowth is also a decolonial agenda, one that focuses on transforming relationships between populations that have been historically and presently exploited by others. Colonialist modes of economic production were (are) grotesque, and their legacies and practices persist. Global frameworks that allow for more equitable use of resources should be pursued^89^. Global exploitation continues and global flows of capital and aid are not simply “good” for recipient countries^99^. Colonialism was an important factor in shaping current global inequalities that drive large-scale extraction of material resources from the Global South and to the Global North and the reciprocal return of waste and emissions from the Global North to the Global South. But simply removing the Global North’s economic production and consumption, with its associated remittance, investment, and trade relationships does not solve a problem created by colonialism.

People may be tempted to dismiss the results of this analysis because the work is model-based. The future is fundamentally unknowable and “all models are wrong,” I fully acknowledge. But I also believe that models help researchers quantify the magnitude of effects and explore dynamics that are hard to anticipate using non-model-based analysis. Also, much of the concern presented by those promoting a degrowth perspective is derived from model-based results, such as those used by the IPCC and original model-based analysis pursued in the *Limits to Growth* work that was so pioneering for so many^40^.

## Conclusion

Radical changes driven by anthropogenic climate change may indeed warrant a radical transformation of how we live. But solutions to global problems should also not create new challenges. If the goal of global development is for humans to live their best lives with a broad range of capabilities while recognizing natural system limitations, then minimizing future human suffering at the hands of climate change should not be achieved by increasing human suffering in the short, medium, and long-term. Balanced solutions require a multipronged approach that embeds markets in society, promotes investment in education, health, and basic human services, develops technologies with a careful eye towards externalities, leverages and invests heavily in science, and promotes governance that is inclusive, transparent, and effective.

### Supplementary Information

Supplementary Information.

## Data Availability

The datasets used and/or analysed during the current study available from the corresponding author on reasonable request. All model results can be reproduced by downloading the International Futures tool at korbel.du.edu/pardee with specific scenarios available here https://ifs02.du.edu/Replication%20Files/DeGrowth%20sce.zip.
