# Supplementary material for: Modeling transformational policy pathways on low growth and negative growth scenarios to assess impacts on socioeconomic development and carbon emissions
Source: Sci Rep. 2023 Sep 25;13:15996. doi: 10.1038/s41598-023-42782-y (PMC10520034; doi:10.1038/s41598-023-42782-y)
Supplement: Supplementary file 1 — Supplementary Information. [file 41598_2023_42782_MOESM1_ESM.docx]

**Appendix**

***Countries in World Bank Income Groups***

| ***High Income Countries*** | ***Low Income Countries*** | ***Low Middle Income Countries*** | ***Upper Middle Income Countries*** |
| --- | --- | --- | --- |
| Australia | Afghanistan | Algeria | Albania |
| Austria | Burkina Faso | Angola | Argentina |
| Bahamas | Burundi | Bangladesh | Armenia |
| Bahrain | Central African Republic | Benin | Azerbaijan |
| Barbados | Chad | Bhutan | Belarus |
| Belgium | Congo, Dem. Republic of the | Bolivia | Belize |
| Brunei Darussalam | Eritrea | Cabo Verde | Bosnia and Herzegovina |
| Canada | Ethiopia | Cambodia | Botswana |
| Chile | Gambia | Cameroon | Brazil |
| Croatia | Guinea | Comoros | Bulgaria |
| Cyprus | Guinea Bissau | Congo | China |
| Czech Republic | Haiti | Cote D'Ivoire | Colombia |
| Denmark | Korea, Dem. People's Republic | Djibouti | Costa Rica |
| Estonia | Liberia | Egypt | Cuba |
| Finland | Madagascar | El Salvador | Dominican Republic |
| France | Malawi | Eswatini | Ecuador |
| Germany | Mali | Ghana | Equatorial Guinea |
| Greece | Mozambique | Honduras | Fiji |
| Hong Kong | Niger | India | Gabon |
| Hungary | Rwanda | Kenya | Georgia |
| Iceland | Sierra Leone | Kyrgyzstan | Grenada |
| Ireland | Somalia | Lao People's Dem. Republic | Guatemala |
| Israel | Sudan | Lesotho | Guyana |
| Italy | Sudan South | Mauritania | Indonesia |
| Japan | Syrian Arab Republic | Micronesia | Iran |
| Korea, Republic of | Tajikistan | Moldova, Republic of | Iraq |
| Kuwait | Togo | Mongolia | Jamaica |
| Latvia | Uganda | Morocco | Jordan |
| Lithuania | Yemen | Myanmar | Kazakhstan |
| Luxembourg |  | Nepal | Kosovo |
| Malta |  | Nicaragua | Lebanon |
| Mauritius |  | Nigeria | Libya |
| Netherlands |  | Pakistan | Macedonia, North |
| New Zealand |  | Palestine | Malaysia |
| Norway |  | Papua New Guinea | Maldives |
| Oman |  | Philippines | Mexico |
| Panama |  | Sao Tome and Principe | Montenegro |
| Poland |  | Senegal | Namibia |
| Portugal |  | Solomon Islands | Paraguay |
| Puerto Rico |  | Sri Lanka | Peru |
| Qatar |  | Tanzania | Russian Federation |
| Romania |  | Timor-Leste | Samoa |
| Saudi Arabia |  | Tunisia | Serbia |
| Seychelles |  | Ukraine | South Africa |
| Singapore |  | Uzbekistan | St. Lucia |
| Slovakia |  | Vanuatu | St. Vincent and the Grenadines |
| Slovenia |  | Viet Nam | Suriname |
| Spain |  | Zambia | Thailand |
| Sweden |  | Zimbabwe | Tonga |
| Switzerland |  |  | Turkey |
| Taiwan |  |  | Turkmenistan |
| Trinidad and Tobago |  |  | Venezuela, Bolivarian Republic |
| United Arab Emirates |  |  |  |
| United Kingdom |  |  |  |
| United States of America |  |  |  |
| Uruguay |  |  |  |

***Scenario Names***

| ***Graph Legend Names*** | ***Scenario Names*** |
| --- | --- |
| Base | IFs Current Path |
| Gr-1 | Global Degrowth |
| Gr-1Gin2.5 | Global Degrowth and Inequality |
| Gr-1HiInc | High Income Degrowth |
| Gr-1HiIncGin | High Income Degrowth and Inequality |
| Gr-1HiIncMil | High Income Degrowth and Military Spending |
| Gr-1HiIncTr | High Income Degrowth and Government Transfers |
| Gr-1HiIncUto | High Income Degrowth and Big Push |
| Gr-1HiMidInc | High Income and Upper Middle Income Degrowth |
| Gr-1HiMidIncGin | High Income and Upper Middle Income Degrowth and Inequality |
| Gr-1HiMidIncMil | High Income and Upper Middle Income Degrowth and Military Spending |
| Gr-1HiMidIncTr | High Income and Upper Middle Income Degrowth and Government Transfers |
| Gr-1HiMidIncUtop | High Income and Upper Middle Income Degrowth and Big Push |
| Gr-1HiMidLow | High Income, Upper Middle Income, Lower Middle Income Degrowth |
| Gr-1HiMidLowGin | High Income, Upper Middle Income, Lower Middle Income Degrowth and Inequality |
| Gr-1HiMidLowMil | High Income, Upper Middle Income, Lower Middle Income Degrowth and Military Spending |
| Gr-1HiMidLowTr | High Income, Upper Middle Income, Lower Middle Income Degrowth and Government Transfers |
| Gr-1HiMidLowUtop | High Income, Upper Middle Income, Lower Middle Income Degrowth and Big Push |
| Gr-1Mil0 | Global Degrowth and Military Spending |
| Gr-1Tr | Global Degrowth and Government Transfers |
| Gr-1Utop | Global Degrowth and Big Push |
| Gr0 | Global No Growth |
| Gr0Gin | Global No Growth and Inequality |
| Gr0HiInc | High Income No Growth |
| Gr0HiIncGin | High Income No Growth and Inequality |
| Gr0HiIncMil | High Income No Growth and Military Spending |
| Gr0HiIncTr | High Income No Growth and Government Transfers |
| Gr0HiIncUtop | High Income No Growth and Big Push |
| Gr0HiMidInc | High Income and Upper Middle Income No Growth |
| Gr0HiMidIncGin | High Income and Upper Middle Income No Growth and Inequality |
| Gr0HiMidIncMil | High Income and Upper Middle Income No Growth and Military Spending |
| Gr0HiMidIncTr | High Income and Upper Middle Income No Growth and Government Transfers |
| Gr0HiMidIncUtop | High Income and Upper Middle Income No Growth and Big Push |
| Gr0HiMidLowm | High Income, Upper Middle Income, Lower Middle Income No Growth |
| Gr0HiMidLowmGin | High Income, Upper Middle Income, Lower Middle Income No Growth and Inequality |
| Gr0HiMidLowmMil | High Income, Upper Middle Income, Lower Middle Income No Growth and Military Spending |
| Gr0HiMidLowmTr | High Income, Upper Middle Income, Lower Middle Income No Growth and Government Transfers |
| Gr0HiMidLowmUtop | High Income, Upper Middle Income, Lower Middle Income No Growth and Big Push |
| Gr0Mil | Global No Growth and Military Spending |
| Gr0Tr | Global No Growth and Government Transfers |
| Gr0Utop | Global No Growth and Big Push |

***Current Path GDP Growth Comparison***

Table 2: GDP growth by World Bank income group by decade using the IFs projection.

|  | **High Income** | **Low Income** | **Low Middle Income** | **Upper Middle Income** |
| --- | --- | --- | --- | --- |
| *2017* | 2.4% | 3.0% | 5.3% | 4.7% |
| *2030* | 1.2% | 5.0% | 4.5% | 3.6% |
| *2040* | 1.2% | 6.7% | 4.7% | 2.9% |
| *2050* | 1.1% | 7.2% | 4.4% | 2.3% |
| *2060* | 1.2% | 6.9% | 4.1% | 2.1% |
| *2070* | 1.2% | 5.8% | 3.8% | 2.0% |
| *2080* | 1.1% | 4.5% | 3.5% | 1.7% |
| *2090* | 1.1% | 3.3% | 3.1% | 1.5% |
| *2100* | 1.2% | 2.4% | 2.7% | 1.3% |

Table 3: IFs global GDP growth by decade compared with SSP scenarios.

|  | **IFs** | **SSP1** | **SSP2** | **SSP3** | **SSP4** | **SSP5** |
| --- | --- | --- | --- | --- | --- | --- |
| *2017* | 3.2% | 3.2% | 3.2% | 3.2% | 3.2% | 3.2% |
| *2030* | 2.3% | 4.8% | 3.7% | 2.8% | 3.8% | 5.8% |
| *2040* | 2.3% | 4.0% | 3.1% | 1.9% | 2.8% | 4.8% |
| *2050* | 2.2% | 3.1% | 2.7% | 1.3% | 2.1% | 3.7% |
| *2060* | 2.2% | 2.4% | 2.4% | 1.2% | 1.6% | 3.1% |
| *2070* | 2.2% | 1.9% | 2.2% | 1.2% | 1.3% | 2.6% |
| *2080* | 2.0% | 1.5% | 2.0% | 1.3% | 1.1% | 2.2% |
| *2090* | 1.9% | 1.2% | 1.9% | 1.3% | 0.9% | 1.9% |
| *2100* | 1.7% | 0.9% | 1.7% | 1.3% | 0.8% | 1.6% |

***Complete Results***

Figure 8: GDP at MER for all scenarios for the World, 2017-2100.

Figure 9: GDP per capita at PPP for scenarios for the World, 2017-2100.

Figure 10: Population for scenarios for the World, 2017-2100.

Figure 11: Deaths by scenario for the World, 2017-2100.

Figure 12: Millions of years of education for people 15 years and older by scenario for the World, 2017-2100.

Figure 13: Millions of people living on less than $1.90 per day (at PPP) for scenarios and the World, 2017-2100.

Figure 14: Military Spending by scenario for the World, 2017-2100.

Figure 15: Health spending by scenarios for the World, 2017-2100.

Figure 16: Education spending by scenario for World, 2017-2100.

Figure 17: Government transfers for pension and welfare by scenarios for the World, 2017-2100.

Figure 18: Gini coefficient for income equality by scenario for World, 2017-2100.
